# Evaluation of an innovative low flow oxygen blender system for global access

**DOI:** 10.3389/fped.2022.981821

**Published:** 2022-09-12

**Authors:** Ellie Ng, Michelle Dundek, Thomas F. Burke

**Affiliations:** ^1^Vayu Global Health Foundation, Boston, MA, United States; ^2^Global Health Innovation Lab, Department of Emergency Medicine, Massachusetts General Hospital, Boston, MA, United States; ^3^Department of Emergency Medicine, Harvard Medical School, Boston, MA, United States; ^4^Department of Global Health and Population, Harvard TH Chan School of Public Health, Boston, MA, United States

**Keywords:** oxygen blender system, global health, hypoxemia, low flow oxygen, oxygen delivery, oxygen concentrator

## Abstract

**Background:**

Safe and effective oxygen delivery methods are not available for the majority of infants and young children globally. A novel oxygen blender system was designed to accurately deliver concentration-controlled, oxygen-enriched air to hypoxemic children up to age five. The system does not require compressed medical air, is compatible with both oxygen tanks and oxygen concentrators, and is low cost. This is the first study that tested the performance of the innovative oxygen blender system.

**Methods:**

The performance of the oxygen blender system was assessed *in vitro* based on delivered oxygen levels and flow rates with an oxygen tank, an oxygen tank using a nasal occlusion model, and an oxygen concentrator.

**Results:**

The measured %O_2_ of the performance test was within ± 5% of full scale (FS) of the target value across all flows and all nasal cannulas. Occlusion testing demonstrated that 50% occlusion did not significantly affect the system outputs. The oxygen blender system was shown to be compatible with both oxygen tanks and oxygen concentrators.

**Conclusions:**

The novel oxygen blender system accurately controls oxygen concentrations and blended air flow rates, and is compatible with both oxygen tanks and oxygen concentrators. This innovation may be an opportunity for improved infant and child oxygen treatment worldwide.

## Introduction

Tens of millions of children worldwide under the age of five suffer an acute respiratory illness (ARI) annually, many of whom would benefit from quality oxygen therapy (1). Of the children that suffer from an ARI nearly ten million meet the World Health Organization classification of pneumonia and one million unnecessarily lose their lives (1). Hypoxemia, or low blood oxygen concentration, is also a common complication of some of the most prevalent causes of newborn mortality, including respiratory distress syndrome (RDS), birth asphyxia, and sepsis (2). Provision of supplemental oxygen can be a lifesaving treatment for hypoxemic infants and children. In Papa New Guinea, implementation of an improved oxygen system resulted in a 35% decrease in deaths of children with pneumonia (3). It is estimated that improved access to oxygen can save the lives of over 120,000 children with pneumonia annually (4).

Access to oxygen has grown over the past decades and is expanding particularly rapidly now due to its role in treating patients with Covid-19. New oxygen plants are being built, increasing the availability of oxygen tanks. Oxygen concentrators are being distributed, providing non-depletable oxygen to hospitals with continuous electricity. While this has led to improved access to oxygen therapy for many hypoxemic children, it has also increased the risk of complications from exposure to excessive amounts of oxygen. Even relatively low flow rates of pure oxygen can lead to hyperoxic lung injury (5). Premature infants are especially vulnerable; hyperoxia in this patient population can cause chronic lung disease, brain injury, and retinopathy of prematurity (ROP) (6). ROP is an especially common and troublesome complication that can lead to blindness. In one Indian hospital, 44% of premature infants screened had ROP (7).

To minimize the risks from exposure to excessive amounts of oxygen, infants and children receiving oxygen therapy should receive a mixture of oxygen diluted with air (8, 9). Pneumatic blenders can be connected to high pressure air and oxygen sources. They mix the two gases to provide oxygen concentrations from 21–100%, allowing providers to titrate delivered oxygen based on the patient's needs. However, these blenders are inaccessible to many hospitals because they are expensive, incompatible with oxygen concentrators, and require compressed medical air, which is not as readily available as compressed oxygen (10). A 2021 survey of sub-Saharan neonatal units found that 56% of neonatal units had no ability to blend air and oxygen, and only 1% had the ability to blend air and oxygen for every patient (11). Hospitals without blending have no choice but to provide 100% oxygen to their patients.

In adults, Venturi masks, which use a jet of high velocity oxygen to entrain ambient air, can be used to inexpensively mix air and oxygen. However, the air/ oxygen blenders in Venturi masks are designed exclusively for use with face masks and are incompatible with nasal cannula (12) which are the preferred method for delivering oxygen to children and infants (2, 13). Venturi blenders on the market are inaccurate and inefficient when attached to higher resistance circuits, especially at the lower flows needed by smaller children and infants (12). There remains an unmet need for an affordable method of providing blended low flow oxygen therapy to children and infants *via* nasal cannula.

In 2020, Mollazadeh-Moghaddam et al. described a Venturi device that could deliver low flows of blended air/oxygen to thin tubing like nasal cannula (14). Similar Venturi devices have been used in CPAP systems before, but not for low flow infant nasal cannula oxygen therapy (15, 16). The design of this Venturi device was improved and integrated into a circuit with a low resistance nasal cannula to create a complete system for delivery of blended air/ oxygen to infants and children up to age five (Vayu Global Health Innovations, Boston, MA, USA). The system can be obtained from Revital Healthcare (EPZ) Limited under the name “Vayu Oxygen Blender System.” These *in vitro* studies were designed to evaluate the outputs of the oxygen blender system.

## Methods

We conducted three separate studies *in vitro*. The first study tested the ability of the oxygen blender system to deliver the target oxygen concentrations and flow rates. The next study tested the performance of the system in nasal models to assess the effect of percent nasal occlusion on the outputs of the device. Finally, the last study tested the ability of the device to deliver the stated flow rates at all oxygen concentrations when powered by an oxygen concentrator.

### The oxygen blender system

The oxygen blender system delivers low flows (0.5–4.0 L/min) of oxygen-enriched air to patients up to age five. It requires an external source of pressurized oxygen with an adjustable flowmeter on the outlet. By controlling the flow rate of oxygen into the blender with the flowmeter, the user also effectively controls the oxygen input pressure. This is due to pressure being proportional to flow rate for the same resistance (the resistance the blender provides).

**Figure 1:**
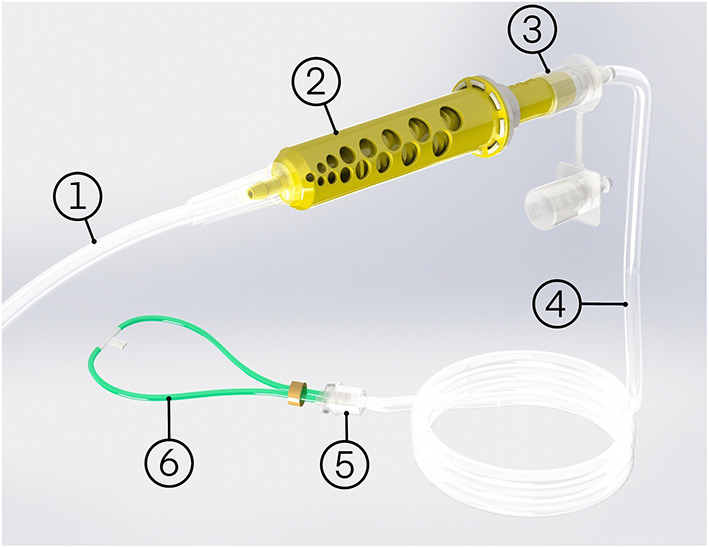
The oxygen blender system comprises of: (1) oxygen tube, (2) oxygen blender system blender, (3) oxygen blender system rotator, (4) patient tubing, (5) cannula adapter, (6) nasal cannula, and an optional oxygen source adapter (not pictured).

The oxygen blender system is shown in Figure 1. Oxygen flows from the source into a Venturi blender. The Venturi blender has been described previously in the context of a bubble continuous positive airway pressure system (16). It generates a mixture of oxygen- enriched air by using a high velocity, low pressure jet of oxygen to entrain ambient air. The mixture is delivered to the patient through nasal cannula. The resistance downstream of venturi blenders is known to affect venturi output. Higher downstream resistances cause a blender to be less efficient (14). Therefore, only cannula with wide tubing and low resistance are compatible with the oxygen blender system. RAM cannulas (Neotech, Valencia, CA, USA) were selected for this study because they are the lowest resistance nasal cannula the authors were aware of (0.446 cmH_2_O/L/min at 2.5 L/min) (17). The system includes four sizes of nasal cannula, and it is recommended to use the size that occludes 50% of the patient's nares.

**Figure 2:**
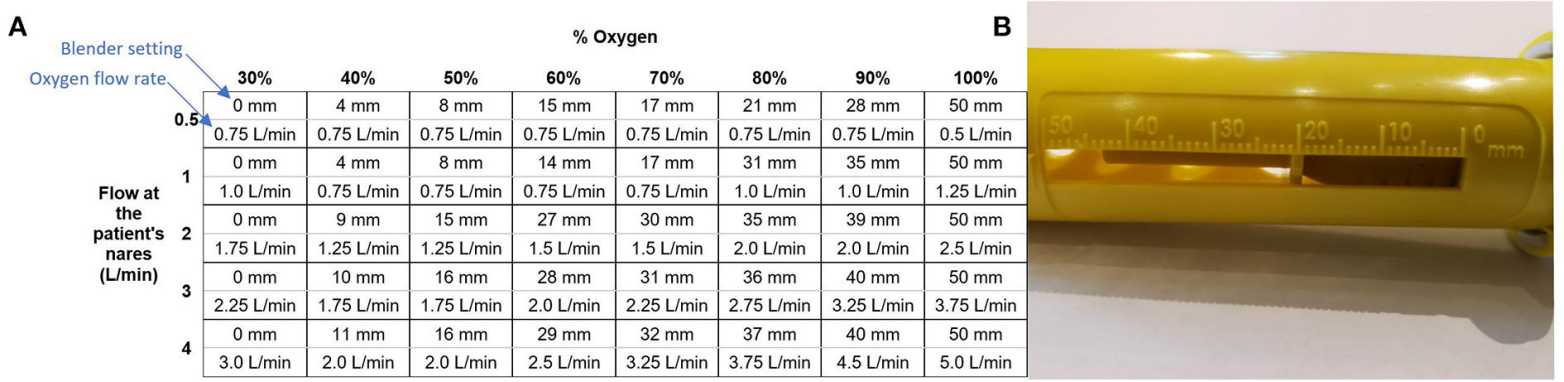
The chart used to set the blender and oxygen flow **(A)** and the scale on the blender set to 20 mm **(B)**.

The flow rate of oxygen into the blender that the user controls is different from the flow rate of blended air and oxygen out of the blender that the patient receives. This is due to the addition of ambient air. The system includes a chart (Figure 2) that is used to determine the oxygen flow rate and blender setting (position of an indicator on the blender along a numbered mm scale) that should be used to achieve the desired blended flow rate and oxygen concentration.

### Performance testing

**Figure 3:**
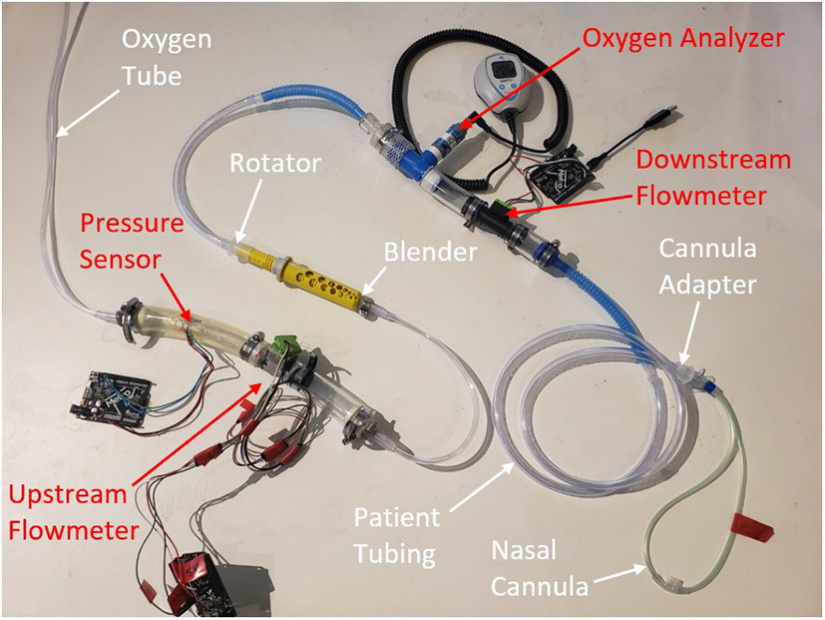
Test set-up for performance testing. The white labels and arrows annotate the oxygen blender system components and the red annotate test sensors.

The testing setup is shown in Figure 3. An oxygen tank with an adjustable outlet pressure was used to run the system. SFM3000 mass flow sensors (Sensirion, Stafa, Switzerland) were placed upstream and downstream of the blender to measure the flow of pure oxygen into the blender and the flow rate of blended air/ oxygen out of the blender, respectively. An MPRLS pressure sensor (Honeywell, Charlotte, NC, USA) was used to monitor the pressure at the outlet of the oxygen source. A maxO_2_+ oxygen analyzer (Maxtec, West Valley City, Utah, USA) inline after the blender measured the oxygen concentration of the oxygen-enriched air.

The oxygen flow rate, blended air/ oxygen flow rate, pressure at the oxygen source, and oxygen concentration of the blended flow were recorded for all combinations of the following variables: cannula sizes S, M, L and XL; 30, 40, 50, 60, 70, 80, 90 and 100% target oxygen concentrations; and target blended flow rates of 0.5, 1.0, 2.0, 3.0 and 4.0 liters per min (L/min). For each data point, the chart was consulted to determine the blender setting in mm and oxygen flow rate to be used. The blender was adjusted to the correct setting and the oxygen flow was increased until the upstream flow sensor indicated that the oxygen flow rate was within 0.05 L/min of the suggested value. The system was given 30 sec to stabilize and then data was recorded. This test was repeated on three separate oxygen blender systems. The blended air/ oxygen flow rate, oxygen flow rate, and oxygen concentration were used to determine the efficiency of the system at different settings. Efficiency was defined as the percentage of input oxygen flow blended and delivered to the nasal cannula.

### Occlusion testing

**Figure 4:**
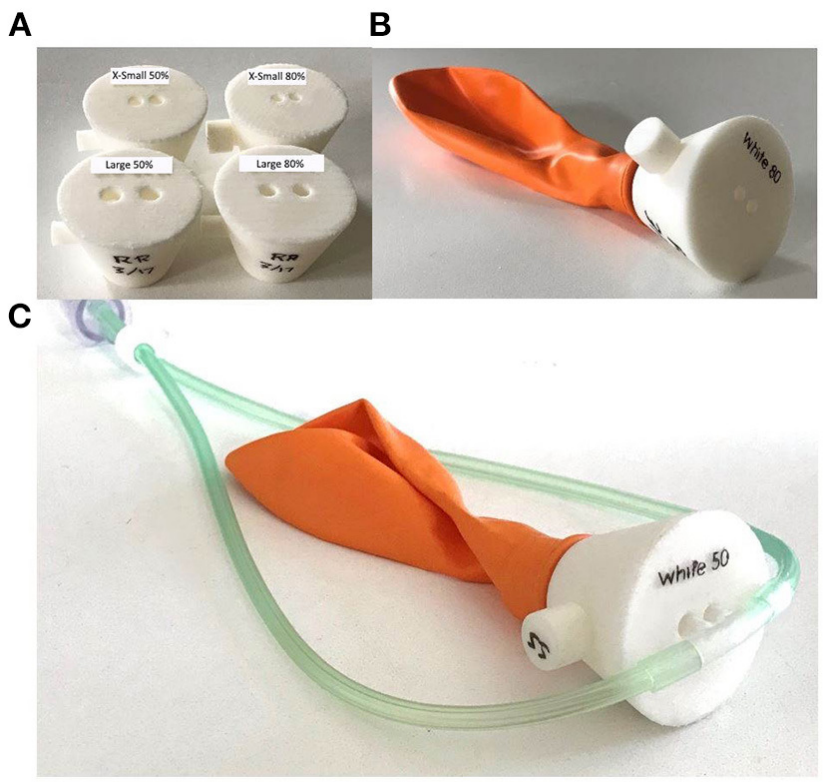
Nasal occlusion models representative of 50 and 80% occlusion for the smallest and largest sizes of nasal cannula **(A)**. Each nasal occlusion model was attached to a balloon creating a closed circuit **(B)** and the nasal cannula were inserted into the nares openings **(C)**.

The same setup was used for occlusion testing as the initial performance tests with the addition of a simple nasal model. Models were created (Figure 4) that corresponded to 50 and 80% occlusion of the nares when used with the smallest and largest nasal cannula sizes, for a total of four models.

The blended air/ oxygen flow rate, pressure at the oxygen source, and oxygen concentration of the blended flow were recorded for all combinations of the following variables: 50% and 80% occlusion, S and XL cannula sizes, 30, 40, 50, 60, 70, 80, 90 and 100% oxygen, and blended flow rates of 0.5, 1.0, 2.0, 3.0 and 4.0 L/min. The oxygen flow rate and blender were set per the chart and data recorded after 30 sec.

### Oxygen concentrator testing

The same setup was used for oxygen concentrator testing as with performance testing except the oxygen tank was replaced with an oxygen concentrator (Jay-10 Dual Flow, Longfian, Baoding, China). The oxygen concentrator was rated to provide 0.5–10 L/min at 6–10 psi (18). The medium sized nasal cannula was used, otherwise the same measurements were taken for the same combination of variables as with the performance testing.

## Results

**Figure 5:**
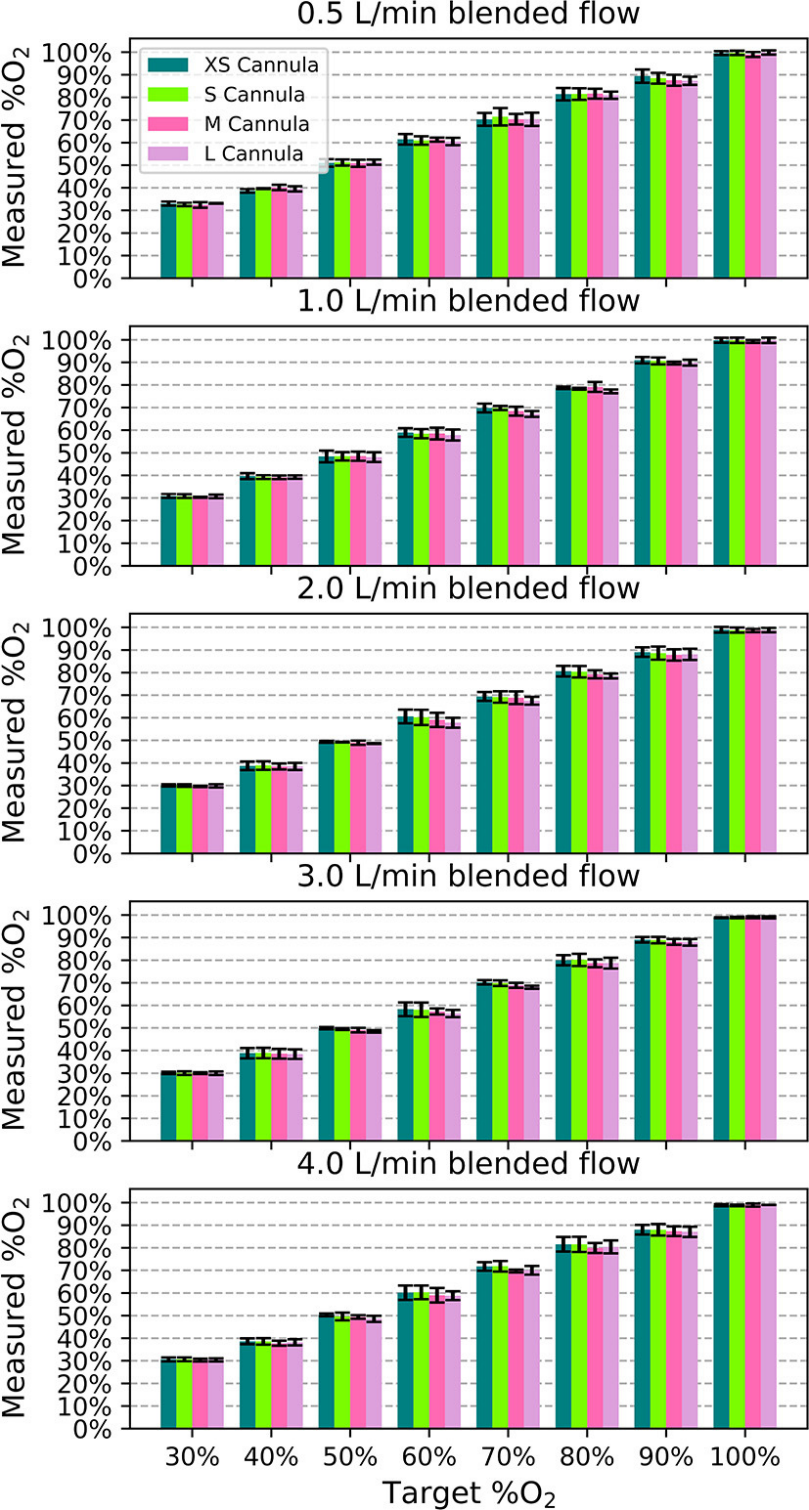
Measured oxygen concentration as a function of target oxygen concentration for each blended flowrate to show the deviation from the intended value. Each bar indicates the mean value across the different samples and the error bars denote standard deviation.

In performance testing, the measured oxygen concentration was within 0.5% of the target oxygen concentration on average. The measured %O_2_ was always within ± 5% of full scale (FS) of the target for all samples, flows, and nasal cannula, as shown in Figure 5 (error bars are standard deviation). Generally, smaller nasal cannula resulted in slightly higher measured oxygen concentrations.

On average, the measured blended flow was within 0.1 L/min of the target blended flow rate. For each cannula size, flow rate and oxygen concentration, the average blended flow rate was within 0.5 L/min of the target blended flow rate. Smaller nasal cannula resulted in slightly lower flow rates. The pressure at the outlet of the tank needed to generate each setting varied from 0.23 psi to 10 + psi (out of sensor range).

**Figure 6:**
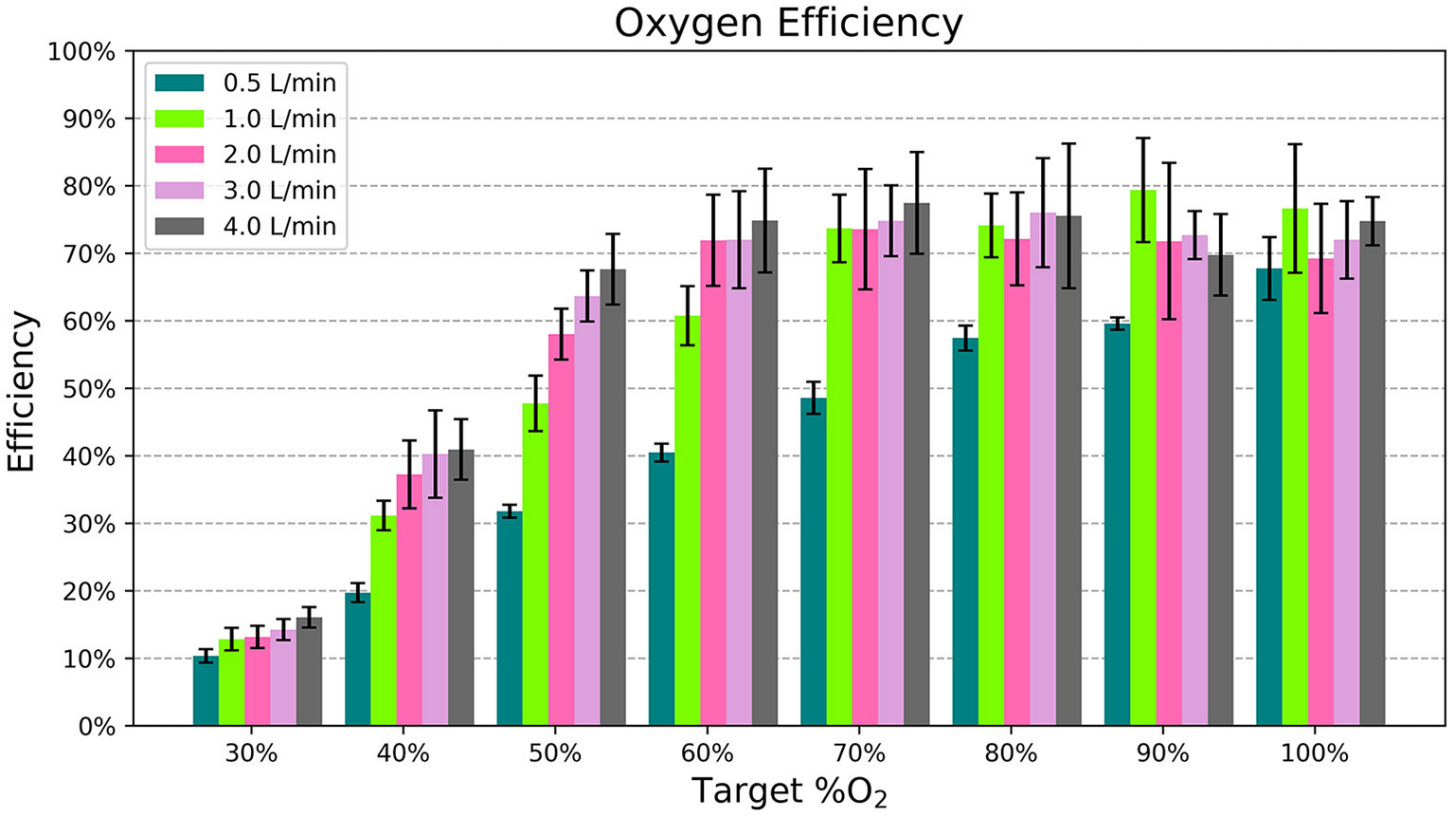
The average efficiency, or percentage of oxygen flow delivered to the patient, for each flow rate and %O_2_ setting. Each bar indicates the mean across different samples and nasal cannula and the error bars denote standard deviation.

Figure 6 displays the efficiency of the blender at different settings. It was more efficient for higher oxygen concentration settings and flows > 0.5 L/min.

**Figure 7:**
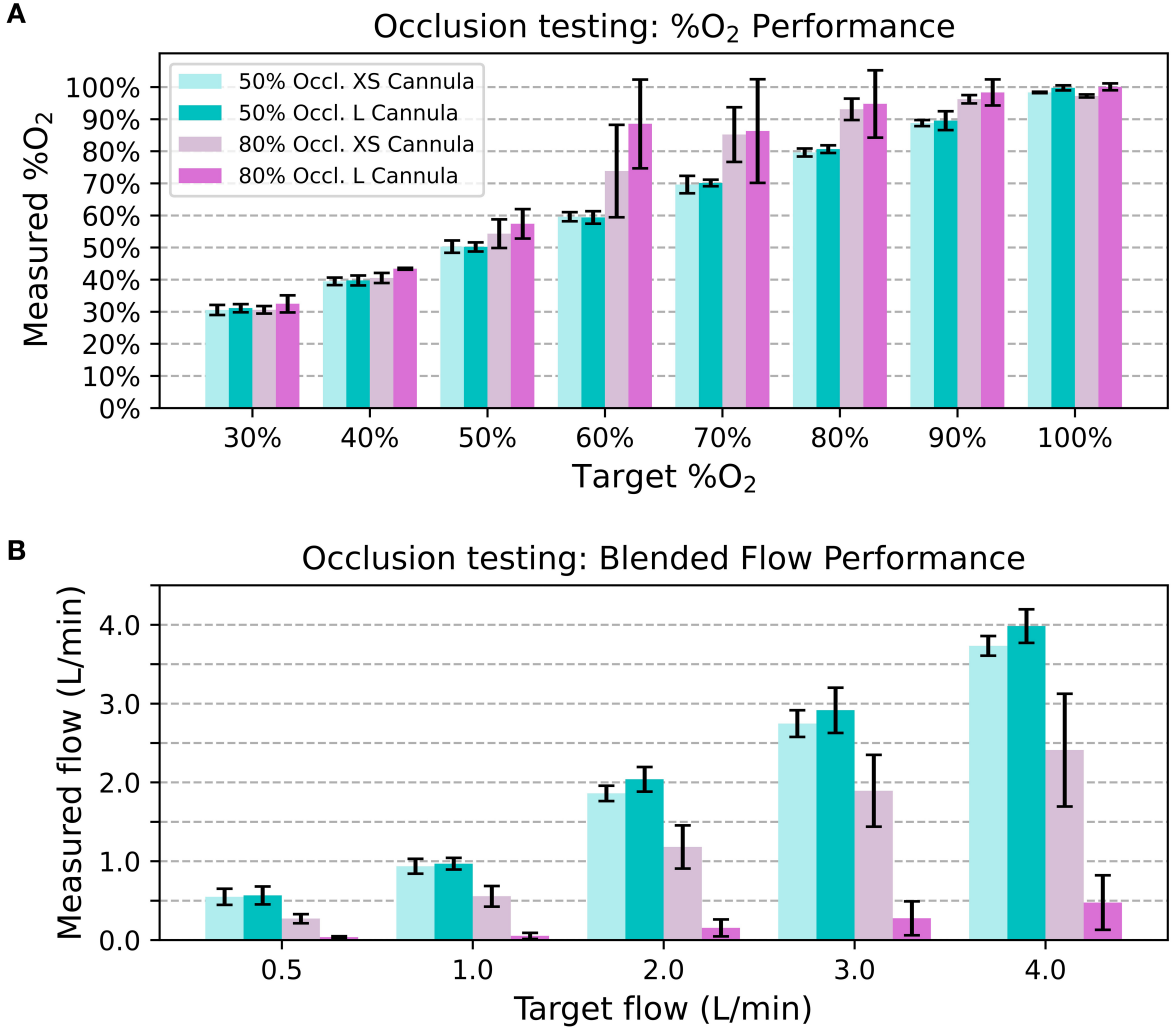
Average measured oxygen concentration in the nasal occlusion models. Each bar indicates the mean across different flow rates and the error bars denote standard deviation **(A)**. Average blended flow rate for different target flow rates in the nasal occlusion models. Each bar indicates the mean across different oxygen concentrations and the error bars denote standard deviation **(B)**.

The 50% occlusion model had little effect on system outputs for both cannula sizes. The measured oxygen concentration was always within 5% FS of the target, and the blended flow was always within 0.5 L/min of the target. However, In the 80% occlusion models 40/ 80 (50.0%) of data points were more than 5% FS above the target oxygen concentrations and 58/80 (72.5%) of data points were more than 0.5 L/min lower than the desired flow. Figure 7 displays the oxygen concentration and flow performance in the different models.

**Figure 8:**
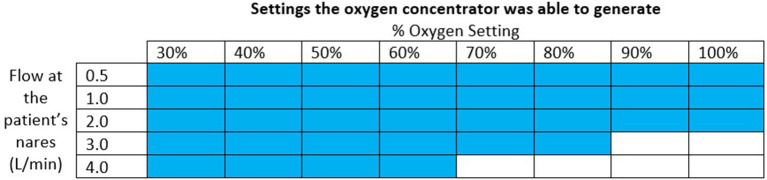
Chart indicating with colored squares which settings the oxygen concentrator was able to generate.

The oxygen concentrator used for the final test was able to supply the oxygen blender system circuit with a maximum of 3.0 L/min of 91% oxygen at 6.5 psi. This was sufficient to generate the settings indicated in Figure 8. The pressure at the outlet of the concentrator ranged from 0.29 psi to 6.51 psi for the settings it was able to generate. The measured oxygen concentration was slightly lower than the target oxygen concentration when the concentrator powered the system.

## Discussion

In low flow oxygen therapy, the amount of oxygen delivered is determined by the flow rate and the oxygen concentration used. Low flow oxygen therapy provides flow rates that are lower than a patient's inspiratory demands. A patient breathes in both supplemental oxygen flow and air from around the nasal cannula. Therefore, low flow oxygen therapy cannot provide oxygen as accurately as occlusive systems where the oxygen concentration of the breathing gases is the FiO_2_. However, it is still important to know both the oxygen concentration and flow rate being delivered to a patient to approximate how much oxygen they are receiving. Oxygen is a drug and high levels are toxic. Hyperoxia causes chronic lung disease, brain injury, and retinopathy of prematurity, especially among vulnerable newborns (6). These *in vitro* tests of the low flow oxygen blender system showed it helps users avoid over oxygenating the patient by delivering accurate flowrates and oxygen concentrations with both oxygen tanks and oxygen concentrators.

The flowrate and resistance downstream of venturi blenders are known to affect venturi output (14). Nasal prongs are the preferred method of low flow oxygen delivery (2, 13) and their resistances vary with size. Even so, amongst all the combinations of cannula sizes, flow rates, and oxygen concentrations, the delivered oxygen concentrations were all within 5% FS of the setting. This is comparable to other commercial blenders with 3% FS tolerances that cost up to $2,750. Furthermore, the delivered flowrate was always within 0.5 L/min of the target flowrate across all settings and cannula sizes.

Oxygen is a valuable resource, so it is important to avoid wasting it. For the most part, the blender's addition of ambient air to the oxygen stream helps conserve compressed gas. For example, according to the chart in Figure 2, it only takes 1.75 L/min of oxygen flow to generate 3.0 L/min of blended flow at 50% oxygen. However, the efficiency of the blender varies depending on the settings used. Efficiency is the lowest at 30% oxygen, where the Venturi blender is open to its widest point and flow can leak out of the blender. Even with this low efficiency, the blended flow generated for a fixed oxygen flow rate ≥ 1 L/min at the 30% setting is still greater than the input oxygen flow. Higher oxygen concentrations showed higher efficiency, around 70%. This efficiency was still <100% due to the loss of flow through the rotator, which necessarily has a leak due to its moving swivel joint. Future iterations could add a gasket to reduce this leak.

Since resistance downstream of the venturi blender affects venturi output, it is also important to consider the effect a patient's nose might have on the delivered flowrates and oxygen concentrations. As seen with the 80% occlusion model, high resistance resulted in lower flowrates and higher oxygen concentrations than expected. Results from testing with the 50% occlusion model were comparable to testing without a nasal model. With the 50% occlusion model, all delivered oxygen concentrations were within 5% of the FS of settings and the delivered flowrates were within 0.5 L/min of the settings. 50% or less occlusion of the nares is recommended, and a size guide that helps users select the correct size nasal prongs is provided with every oxygen blender system.

**Figure 9:**
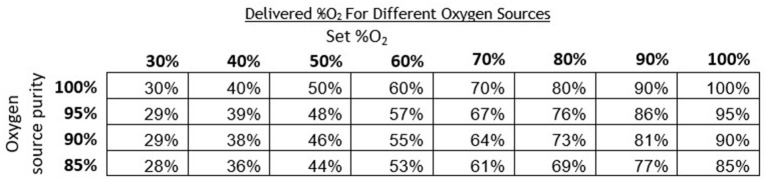
Chart indicating the corrected theoretical output oxygen concentrations for oxygen sources of <100% purity.

Oxygen concentrators are another common oxygen source and typically provide oxygen concentrations in the range of 87–95% (19). Since oxygen concentrators do not provide 100% oxygen, measured O_2_ concentrations with the concentrator were lower compared to measurements taken with a 100% oxygen tank source. The instructions for the oxygen blender system include a chart that provides an adjusted delivered O_2_ concentration per the concentration of the oxygen source (Figure 9). This chart can also be referenced when oxygen cylinders filled by pressure or vacuum swing absorption are used, which have purity <100%.

When connected to an oxygen concentrator, the oxygen blender system was able to provide all settings except oxygen settings of 90% + at 3.0 L/min and 70% + at 4.0 L/min. At lower %O_2_ settings, the blender entrains in a high proportion of ambient air and adds it to the oxygen flow, resulting in a blended flowrate that is greater than the oxygen flowrate. At higher %O_2_ settings, the blender entrains less ambient air and thus needs higher oxygen flowrates—which correspond to higher oxygen source pressures—to attain the same blended flowrates. Because oxygen concentrators have lower outlet pressures than tanks or central supplies, the oxygen concentrator in this study was unable to provide the maximum flows with the maximum pressures. These high flowrates at high oxygen concentrations are rarely used (20). If a patient requires 100% unblended oxygen at higher flows, therapy can be administered without the blender. The nasal cannula is designed with a barbed adapter that attaches directly to oxygen tubing.

Different oxygen concentrators have different maximum outlet pressures and thus achieve different maximum oxygen flowrates when attached to the oxygen blender system. The oxygen concentrator in this evaluation was selected as a worst-case scenario because it had the lowest maximum flowrate and outlet pressures out of the Newborn Essential Solutions and Technologies (NEST360) recommended concentrators (21). Higher outlet pressures of 20 PSI are common amongst oxygen concentrators that provide 8-10 L/min of oxygen flow which is enough pressure to generate all flows for all %O_2_ settings when connected to the oxygen blender system (19). Notably, the concentrator tested only provided a maximum of 3 L/min at 6.5 psi when it was rated to provide up to 10 L/min at 6-10 psi. The limitation of flow to only 3 L/min can be attributed to the resistance of the oxygen blender restricting the flow based on the maximum outlet pressure. However, the maximum outlet pressure of 6.5 psi was on the lower end of the manufacturer's specifications.

Our study did have some limitations. Further testing using a lung simulator may better characterize the effects a patient's breathing cycle has on the outputs of the oxygen blender system. Testing was only conducted with one oxygen concentrator so future tests with more concentrators could more broadly characterize the oxygen blender system's performance with concentrators of different outlet pressures. While RAM cannulas are often used in low resource settings in the authors' experience, they can be cost prohibitive at over $10 per cannula. Additional tests with a variety of cannulas might identify other compatible cannulas that are more cost effective. Our team has recently developed a low cost and low resistance nasal cannula for the oxygen blender system.

## Conclusion

This novel oxygen blender system accurately delivers target flows and oxygen concentrations when used with a 50% occlusive nasal prong model. It is accurate with the most commonly used flowrates and oxygen concentrations when connected to oxygen tanks and oxygen concentrators.

## Data availability statement

The raw data supporting the conclusions of this article will be made available by the authors, without undue reservation.

## Author contributions

EN and MD provided input on the design of the studies. MD assisted in acquiring measurements and data analysis. TB oversaw all aspects of this research. Literature search, study design, data analysis and manuscript preparation were done by all the authors. All authors contributed to the article and approved the submitted version.

## Funding

This study was funded by Unitaid via Unitaid Explore Grant. Unitaid is a hosted partnership of the World Health Organization. The Vayu Global Health Foundation provided the oxygen blender systems for this study.

## Conflict of interest

Authors MD and EN are engineers with Vayu Global Health Innovations, a public benefit company that develops high quality ultra—low cost oxygen delivery solutions.

The remaining authors declare that the research was conducted in the absence of any commercial or financial relationships that could be construed as a potential conflict of interest.

## Publisher's note

All claims expressed in this article are solely those of the authors and do not necessarily represent those of their affiliated organizations, or those of the publisher, the editors and the reviewers. Any product that may be evaluated in this article, or claim that may be made by its manufacturer, is not guaranteed or endorsed by the publisher.
